# Urinary Incontinence in Active Female Young Adults: Healthcare Preferences, Priorities and Experiences

**DOI:** 10.1007/s00192-024-05786-4

**Published:** 2024-05-07

**Authors:** Rebecca L. Newark, Rachel Thompson

**Affiliations:** Sydney School of Health Sciences, Faculty of Medicine and Health, The University of Sydney, Sydney, Australia

**Keywords:** Athletes, Decision making, Help-seeking behaviour, Surveys and questionnaires, Urinary incontinence

## Abstract

**Introduction and Hypothesis:**

There is a paucity of evidence on the healthcare preferences of active female young adults with urinary incontinence (UI). This research is aimed at examining the population’s healthcare preferences and priorities and their characteristics and experiences to improve access to and use of services.

**Methods:**

This cross-sectional online survey study used a convenience community sample. Participants resided in Australia, were 18–30 years old, had self-reported engagement in physical activity of any frequency and self-reported urine leakage in the previous 4 weeks and met other eligibility criteria. Data were analysed using descriptive analyses.

**Results:**

Thirty-nine participants took part in the study. The majority engaged in recreational exercise (74.2%) and experienced UI of slight to moderate severity (82.9%). Participants preferred to access information and support anonymously online (71.4%) from general practitioners (58.8%), medical specialists (50.0%) or physiotherapists (44.1%). All participants wanted to be involved in the UI management decision-making process. Participants prioritised knowing information over cost (38.2%), changes to daily habits (35.3%) and potential risks or side effects (23.5%) when making decisions about management of UI.

**Conclusion:**

The results highlight the diversity among active female young adults with UI. They emphasise the need for shared decision making and highlight key information needs, providing the basis for the development of decision-making tools and protocols specific to this population. They demonstrate the need for anonymous online information and support, and draw attention to the presence of UI among young recreational exercisers, highlighting the importance of ensuring that information and support is available within this demographic.

**Supplementary information:**

The online version contains supplementary material available at 10.1007/s00192-024-05786-4

## Introduction

Urinary incontinence (UI)—involuntary loss of urine [1]—is common in older, parous women. There are also several international systematic reviews documenting the high prevalence of UI among active young women [2–5]. The studies within these reviews, and other literature generally focuses on elite athletes [6]. Existing research focuses on stress UI [2–5]. However, urge UI and mixed UI have also been observed among active young women [7, 8]. Stress UI refers to the involuntary loss of urine during physical exertions such as sporting activities or sneezing and coughing [1], and it is associated with increased intra-abdominal pressure [2]. Urge UI refers to the involuntary loss of urine associated with a sudden, compelling desire to void, and mixed UI refers to a mix of both urgency and stress UI symptoms [1]. There is a greater documented prevalence of UI in high-impact sports such as trampolining and volleyball [2, 3, 5].

Urinary incontinence can negatively affect the emotions of active young women through embarrassment, fear, and worry, as well as negatively affecting their performance and engagement in physical activity [9, 10]. Despite this, as little as 6% of this population have discussed their incontinence with a healthcare professional [11]. Although active young women are interested in learning more about the prevention and treatment of UI [11], they are often overlooked in UI service delivery and research. Furthermore, the existing research among this population focuses on those performing at the elite level, rather than those participating in physical activity recreationally [6].

Owing to the hesitance of active young women to seek help for UI, as well as the embarrassment and lack of knowledge among the population, it is important that there is improved access to information and support. To the best of our knowledge there is currently no evidence on the preferred person and communication method of the population for accessing information and support with regard to urinary incontinence. Improving knowledge on their healthcare preferences may help to develop resources that can increase the use of healthcare services and improve the delivery of care.

We are also not aware of any evidence of the preferred role of active young women in decision making or their information priorities for decision making about UI management. Research among older women receiving urogynaecological care has demonstrated a preference for active or collaborative roles in the decision-making process [12, 13] and has shown that patients have complete satisfaction with treatment if their physicians are aware of their decision-making needs, highlighting the importance of understanding patient preferences [13]. The current absence of knowledge on patient values in this population is a barrier to providing management counselling through shared decision making, ensuring that patients receive the best management.

This study was aimed at addressing the above-described gaps by generating evidence on the preferences and priorities of incontinent active young women for information support and decision making, as well as their characteristics and experiences regarding UI and physical activity.

## Materials and Methods

### Study Design and Participants

This study used a cross-sectional survey design. Individuals meeting the following criteria were eligible to participate: female sex at birth, 18–30 years old, confident in reading and writing in English, residing in Australia, self-reported UI within the past 4 weeks, and self-reported engagement in physical activity of any frequency. Participation in the study was voluntary and anonymous. A browser cookie system prevented participants from submitting multiple responses while maintaining anonymity.

### Recruitment and Consent

This study used convenience community sampling. Participants were recruited through advertisements on Facebook, Instagram, Twitter and research websites (see Supplementary Material 1). Relevant sporting organisations and university student societies were contacted with information regarding the study. Flyers were placed in relevant locations, such as gymnasiums, in the large metropolitan city where the researchers were located. All advertisements provided a link and/or QR code for a website that displayed the Participant Information Statement (see Supplementary Material 2). This allowed participants to provide informed, electronic consent to the research and complete the survey. Participants’ eligibility was confirmed through a series of screening questions at the beginning of the survey. If participants did not meet the eligibility criteria, they could not proceed further in the survey. Recruitment strived for the maximum possible sample size within the available time period.

### Data Collection and Survey

The survey was administered between the 23 April 2023 and the 27 September 2023 through the Qualtrics online survey platform. The survey consisted of 25 questions from five domains, displayed over 16 pages (see Supplementary Material 2). Participants were unable to review or change their answers after submitting a page. Before submitting the page, participants were notified if they had not answered all questions. However, owing to the sensitive nature of the topic, they could continue the survey regardless to maximise retention. If participants did not complete the survey in one sitting, their responses were stored for 1 week. After 1 week all incomplete surveys were submitted to the data set.

Although validated items were used or adapted wherever possible, the survey also contained newly developed items. The survey was informally pretested among five individuals with similar demographics to the study group to ensure technical functionality and that questions were acceptable and understood as intended. Feedback from pretesting was used to refine the wording of questions before the survey was administered.

### Measures

#### Participant Characteristics and Physical Activity Characteristics

Participants reported their age, sex, postcode, Aboriginal or Torres Strait Islander status, language(s) spoken at home, previous pregnancies/births, and health literacy [14]. Participants’ postcode was used to derive two new variables (state of residence and rurality) according to the Modified Monash Model [15]. If a postcode was not included in the Modified Monash Model or had multiple rurality classifications it was excluded from the variables. Health literacy was assessed using a validated single question [14], and other characteristics were assessed using newly developed items. Participants’ physical activity was assessed through newly developed items that asked about type, impact level and competitive level. The impact level was self reported using a visual analogue scale from 0 (“low-impact”) to 10 (“high-impact”). The scale was accompanied by examples of low-impact (“things like walking or swimming”) and high-impact (“things like volleyball or gymnastics”) activities that were adapted from de Mattos Lourenco et al. [2].

#### Urinary Incontinence Characteristics and Experiences

Urinary incontinence was measured using the International Consultation on Incontinence Questionnaire-Urinary Incontinence Short Form (ICIQ-UI SF) [16]. This four-item measure assesses the average severity, impact and type of UI over the past 4 weeks and has been validated to measure and diagnose UI among multiple populations [16]. Participants’ UI severity (slight, moderate, severe, very severe) was determined according to Klovning et al. [17], whereas UI type (mixed, urge, stress) was determined according to Espuña-Pons et al. [18].

A single-item measure from the Australian Longitudinal Study on Women’s Health [19] was used to measure whether participants had previously disclosed their UI to a healthcare professional. This was followed by a newly developed open-ended question on previous management: “What, if anything, have you tried or used to manage your leaking urine?”

#### Preferences and Priorities for Information Support and Decision Making

The questions measuring participants’ preferences and priorities for information support and decision-making were adapted from previous research in other populations [20–22]. The response options for these questions were informed by the same previous studies, as well as UI clinical guidelines and scoping reviews [23, 24].

Participants’ preferences and priorities regarding information and support were measured through two questions. The first question asked about the people from whom or places from which they would like to receive information and support about leaking urine. Participants could select multiple preferences from a list of seven or were able to offer additional preferences not listed. They were then asked to select their top three priority response options. This forced participants to prioritise options, addressing possible ceiling effects observed in previous similar studies, where participants had rated all response options as important [25]. The second question asked participants about their preferred communication method for receiving information and support. Similarly, participants were able to select their preferred communication methods from seven options or disclose other options.

Participants’ preferences and priorities regarding decision-making were measured using a pair of items. The first item measured the information needs for decision-making about the management of UI. Participants were provided with a list of 20 potential patient questions regarding management options and were asked to preference all questions they would want to know about. They were also able to volunteer other questions that were not listed. They were then asked to prioritise their top three highest priority questions, again addressing any possible ceiling effects [25]. The second item used the Control Preferences Scale to assess the degree of control the participant wanted to assume in decision making about managing their UI [26]. This widely adopted measure has been validated within similar populations [12].

#### Open-Ended Responses

The last survey item allowed participants to share any additional information about their experiences or opinions in an open-ended format.

### Analysis

Statistical analysis of the data was performed independently by both authors to ensure accuracy, using the Statistical Package for Social Sciences (SPSS) Version 29. Incomplete questionnaires were included in the analysis. All missing data were presumed to be missing not at random and excluded from analysis of the corresponding variable. Descriptive statistics were used to summarise participant characteristics, including UI and PA. Means and standard deviations were used for continuous variables and counts/percentages were used for categorical variables. Health care preferences and priorities were analysed according to the counts/percentages of participants who selected each response option as a preference and the count/percentage of participants who selected each response option as a top three priority. Data from the open response items were coded inductively, sorted into concepts, and summarised or expressed quantitatively as counts.

## Results

### Participants

There were 39 participants in the eligible sample that was used for analysis. Sixty-one participants began the survey study but 22 were ineligible. One participant did not feel confident reading and writing in English and one did not answer this question. Two participants were older than 30, and 8 did not disclose their age. Five participants were assigned male sex at birth and five participants did not live in Australia. There was a completion rate of 77% as 30 out of the 39 eligible participants finished the survey.

### Participant Characteristics and Physical Activity Characteristics

Participants had a range of demographic characteristics (Table 1). Participants also engaged in a variety of physical activities (Table 2). Most participants engaged in recreational medium-impact activities such as weight training, general exercise and running.

**Table 1:** Participant characteristics

	*n* (%) or , mean ± SD
State of residence (*N* = 38)	
New South Wales	31 (81.6)
Victoria	4 (10.5)
Queensland	2 (5.3)
Australian Capital Territory	1 (2.6)
Rurality of residence (*N* = 36)	
Metropolitan areas (MM1)	35 (97.2)
Regional centres (MM2)	0 (0.0)
Large rural towns (MM3)	1 (2.8)
Medium rural towns (MM4)	0 (0.0)
Small rural towns (MM5)	0 (0.0)
Remote communities (MM6)	0 (0.0)
Very remote communities (MM7)	0 (0.0)
What language(s) do you speak at home? (*N* = 30)	
English	27 (90.0)
Other^a^	3 (10.0)
Health literacy (*N* = 30)	
Limited	3 (10.0)
Adequate	27 (90.0)
Which of the following describes you? (*N* = 30)	
Aboriginal	0 (0.0)
Torres Strait Islander	0 (0.0)
None of the above	30 (100.0)
Which of these apply to you? (*N* = 30)	
I have been pregnant	0 (0.0)
I have had one or more babies	0 (0.0)
None of the above	30 (100.0)
Age (years) (*N* = 39)	21.9 ± 2.6

*SD* standard deviation

^a^Other languages spoken were French/Dutch, Hindi and Mandarin

**Table 2:** Physical activity characteristics

	*n* (%)
How often do you do physical activity, exercise or sport? (*N* = 39)	
Daily	14 (35.9)
A few times a week	23 (59.0)
A few times a month	1 (2.6)
Less than a few times a month	1 (2.6)
Which of these best describes the physical activity, exercise or sport you do most often? (*N* = 31)	
Recreational	23 (74.2)
Regional	6 (19.4)
State	1 (3.2)
National	1 (3.2)
Elite (paid)	0 (0.0)
What type of physical activity, exercise or sport do you do most often?^a^ (*N* = 39)	
Resistance weight training	11 (28.2)
General exercise^b^	11 (28.2)
Running	9 (23.1)
Walking	6 (15.4)
Contact team sports^c^	4 (10.3)
Noncontact team sports^d^	4 (10.3)
Sprinting	2 (5.1)
Cycling	2 (5.1)
Swimming	2 (5.1)
Pilates	2 (5.1)
Triathlon	2 (5.1)
Muay Thai	1 (2.6)
Dance	1 (2.6)
What is the impact level of physical activity, exercise or sport you do most often? (*N* = 31)	
0–3	7 (22.6)
4–6	11 (33.5)
7–10	13 (41.9)

^a^Participants were able to report more than one type of activity

^b^General exercise included “gym”, cardiovascular training, elliptical, high-intensity interval training, group and home exercises

^c^Contact team sports included soccer and rugby league

^d^Non-contact team sports included volleyball and netball

### Urinary Incontinence Characteristics and Experiences

Participants had diverse characteristics and experiences regarding their UI (Table 3). Most participants experienced UI of slight or moderate severity and disclosed some form of previous management, despite not having previously spoken to a healthcare professional.

**Table 3:** Urinary incontinence characteristics and experiences

	*n* (%)
Severity (*N* = 35)	
Slight (1–5)	13 (37.1)
Moderate (6–12)	16 (45.7)
Severe (13–18)	6 (17.1)
Very severe (19–21)	0 (0.0)
Type (*N* = 35)	
Stress urinary incontinence	15 (42.9)
Mixed urinary incontinence	11 (31.4)
Urge urinary incontinence	6 (17.1)
No applicable category^a^	3 (8.6)
How often you do leak urine? (*N* = 39)	
About once a week or less often	21 (53.8)
Two or three times a week	13 (33.3)
About once a day	1 (2.6)
Several times a day	3 (7.7)
All the time	1 (2.6)
How much urine do you usually leak? (*N* = 35)	
A small amount	29 (82.9)
A moderate amount	4 (11.4)
A large amount	2 (5.7)
When does urine leak?^b^ (*N* = 35)	
Leaks when you are physically active/exercising	25 (71.4)
Leaks before you can get to the toilet	17 (48.6)
Leaks when you cough or sneeze	14 (40.0)
Leaks when you have finished urinating and are dressed	6 (17.1)
Leaks for no obvious reason	6 (17.1)
Leaks when you are asleep	5 (14.3)
Leaks all the time	1 (2.9)
Have you ever talked to a health professional about your leaking urine? (*N* = 35)	
Yes	13 (37.1)
No	22 (62.9)
What, if anything, have you tried or used to manage your leaking urine?^b^ (*N* = 27)	
Pelvic floor muscle training	9 (33.3)
Nothing	8 (29.6)
Wearing a liner or pad	7 (25.9)
Increased toileting	6 (22.2)
Lifestyle modifications^c^	3 (11.1)
Medications	2 (7.4)
Avoidance of precipitating activity	2 (7.4)
Botox injections	1 (3.7)
Reduced fluid intake	1 (3.7)
How much does leaking urine interfere with your everyday life? (*N* = 35)	
0–3	18 (51.4)
4–6	10 (28.6)
7–10	7 (20.0)

^a^One participant reported experiencing leakage only when asleep and 2 participants reported experiencing leakage for no obvious reason

^b^Participants were able to disclose more than one option

^c^Modifications included activity modifications (e.g. ocean swimming rather than pool swimming), clothing modifications (e.g. wearing darker clothing) and modifications to the home environment (e.g. waterproof bed sheets)

### Preferences and Priorities for Information Support and Decision-Making

Participants had a range of preferences and priorities for receiving information and support about leaking urine (Table 4). Participants preferred to receive information and support anonymously online from general practitioners (primary care physicians), medical specialists or physiotherapists.

**Table 4:** Preferences and priorities for information support

	Selected as a preference, *n* (%)	Selected as a top three priority, *n* (%)
Which people or places would you like to receive information and support about leaking urine from? (*N* = 34)		
General practitioner	20 (58.8)	18 (52.9)
Medical specialist (e.g. urogynaecologist)	17 (50.0)	14 (41.2)
Physiotherapist	15 (44.1)	15 (44.1)
A website	14 (41.2)	10 (29.4)
Social media	12 (35.3)	8 (23.5)
Sports coach	8 (23.5)	6 (17.6)
Family or friend	6 (17.6)	2 (5.9)
Another health professional/other^a^	4 (11.8)	2 (5.9)
None of these	2 (5.9)	–
How would you like to receive information and support about leaking urine?^b^ (*N* = 35)		
Online, without giving my name	25 (71.4)	
In person, one-on-one	18 (51.4)	
Text message	8 (22.9)	
Telephone call	4 (11.4)	
Online, giving my name	4 (11.4)	
In person, in a group	3 (8.6)	
Video call	1 (2.9)	
Other^b^	1 (2.9)	

^a^Other included exercise physiologists, gynaecologists, sports doctors and psychologists

^b^Other: participant did not elaborate

Participants also had a range of preferences and priorities for decision making about the management of UI (Table 5). Participants’ top preferences and priorities for decision making were information about changes to daily habits and cost. Other information important to participants included risks of side effects, research evidence, efficacy and privacy. All participants preferred an active or collaborative role in decision making, with 71% preferring to make the final decision after seriously considering their healthcare professional’s opinion.

**Table 5:** Preferences and priorities for decision making

	Selected as a preference, *n* (%)	Selected as a top three priority, *n* (%)
There are lots of options for managing leaking urine. If you were deciding how to manage your leaking urine, what would you want to know about each option? (*N* = 34)		
Does it involve changing my habits or activities (e.g. how much coffee I drink)?	23 (67.6)	13 (38.2)
How much does it cost?	23 (67.6)	12 (35.3)
Will it stop me leaking urine completely?	21 (61.8)	5 (14.7)
Will it reduce how often or how much I leak urine?	20 (58.8)	7 (20.6)
Has it been properly studied?	20 (58.8)	5 (14.7)
How long does it take to start working?	17 (50.0)	1 (2.9)
What are the risks or side effects?	16 (47.1)	8 (23.5)
Does it involve a health professional touching inside or near my vagina?	15 (44.1)	7 (20.6)
Does it involve having surgery (an operation)?	15 (44.1)	5 (14.7)
Does it require regular visits to a health professional?	15 (44.1)	4 (11.8)
Does it involve me putting something into my vagina?	15 (44.1)	2 (5.9)
Will other people know that I am using it?	14 (41.2)	7 (20.6)
Does it involve seeing a health professional face to face?	14 (41.2)	2 (5.9)
Can I stop using it if I change my mind?	13 (38.2)	4 (11.8)
How does it work?	12 (35.3)	6 (17.6)
Does it involve a device being put in my body?	11 (32.4)	1 (2.9)
Does it involve having a needle?	10 (29.4)	1 (2.9)
Could it make my leaking urine worse?	9 (26.5)	1 (2.9)
Does it involve staying in a hospital?	9 (26.5)	0 (0.0)
Can I access it online?	6 (17.6)	0 (0.0)
Other^a^	1 (2.9)	0 (0.0)
If you were deciding how to manage your leaking urine, how would you want the final decision to be made? (*N* = 31)		
I would want to make the final decision	4 (12.9)	
I would want to make the final decision after seriously considering my health professional’s opinion	22 (71.0)	
I would want to share responsibility for the final decision with my health professional	5 (16.1)	
I would want my health professional to make the final decision after seriously considering my opinion	0 (0.0)	
I would want my health professional to make the final decision	0 (0.0)	

^a^One further participant used this opportunity to reiterate the importance of knowing cost information

### Open-Ended Responses

Six participants provided open-ended responses in the final survey item, “Please use this space if there is anything else you would like to tell us about your experiences or opinions”. Three participants spoke directly about feelings of embarrassment or shame related to UI. Generally, participants felt that incontinence was a “stigmatised condition” and that as young women they were “embarrassed or ashamed to seek medical help”. One participant spoke about having ceased professional sport to undergo pelvic floor rehabilitation and expressed fear about the implications of future pregnancies on their incontinence.

## Discussion

This study was aimed at determining the preferences and priorities of active young women with regard to information support and decision making, as well as their characteristics and experiences concerning UI and physical activity. All participants in this study wanted to be involved in the decision-making process for the management of UI, highlighting the need for shared decision making between practitioners and patients. This supports similar research showing that the majority of women seeking care for pelvic floor disorders wanted an active or collaborative role in the decision-making process [12, 13], with only 7–9% of patients wanting to play a passive role in decision making. This understanding of participants’ decision-making preferences can be used by clinicians to improve patient satisfaction [13] through promoting shared decision making. Shared decision-making aids may also be appropriate for this population as they want to be involved in the decision-making process, whilst also desiring privacy. The future development of shared decision-making aids for this population should prioritise information on changes to daily habits, cost, potential risks or side effects and efficacy as these were the most widely endorsed information needs of the sample.

The majority of participants preferred to receive information and support anonymously online. Questions about the discretion of different management options were also highly prioritised by participants. This calls attention to the need for increased anonymous online resources about UI tailored to this population. These educational resources should be developed by health professionals as they were the most preferred and prioritised individuals for information and support. These resources could help to improve the population’s knowledge to increase healthcare access among those with UI [4], and potentially be preventative among those without UI [11]. Furthermore, they may be helpful in increasing awareness and decreasing the stigma and embarrassment associated with UI. This embarrassment was somewhat evident within our sample. Although the average effect of UI on participants’ quality of life was modest (4.1 out of 10), there was significant variance between participants, with the effect ranging from 0 to 10 out of 10. Furthermore, the effect was salient enough that some participants disclosed the stigma, shame and embarrassment they felt surrounding their UI within the open-ended questions, similar to other research [9, 10]. These discrepancies may be due to differences in the severity of incontinence, as previous research has shown that active young women who experienced more frequent incontinence reported higher levels of frustration, worry and embarrassment [27]. Regardless, online resources may help to decrease the stigma surrounding the condition and improve the use of healthcare services.

The result of the study also provide valuable insight into the population’s characteristics. Previous research on UI in young people has focused on elite athletes [6]. In contrast, our broader inclusion criteria facilitated a sample of mainly recreational exercisers. This is reflected in the activities that the sample engaged in, including weight training, general “gym” exercise and running. The average impact level of these activities was rated by participants to be 5.8 out of 10. This contrasts with previous research that has focused on the incidence of UI within high-impact sports [2, 3, 5]. Similar to some other research in young adults, participants experienced a mix of stress, urge and mixed UI symptoms [7, 8].

These characteristics and other results from this study have implications for health professionals providing information and support for UI. First, clinicians need to ensure that information and support for UI is available for recreational exercisers participating in low-impact activities, and that urge UI symptoms are common among this population. Second, the prioritisation of discretion and privacy within this population calls into question the appropriateness of universal screening for UI among this population. Future research could assess the acceptability of screening for UI among young recreational exercisers. Nonetheless, these findings do highlight the importance of conducting any questioning in a sensitive and appropriate manner, similar to previous research [28]. Third, it is recommended that information and support be provided by general practitioners, physiotherapists and medical specialists, as they were deemed the most acceptable sources by participants. Finally, this research also highlights the information needs of the population. These information needs could be the focus of efforts to help improve clinicians’ knowledge and confidence when providing information and support, which has previously been identified as a barrier [29]. The results from this study in combination with pre-existing screening tools [28, 30] and clinical guidelines [23, 24] can be used by clinicians to help to facilitate proper access to information and support.

This study is to our knowledge the first of its kind to investigate the healthcare preferences and priorities of active female young adults with UI. It provides valuable insights into the preferences and experiences of some young people with UI and demonstrates the feasibility of examining healthcare preferences within this population. However, this research has used a small convenience sample and recruitment focused particularly on university students in one large metropolitan city in Australia. Hence, the healthcare preferences, priorities and experiences of this sample may not reflect those of all active female young adults with urinary incontinence. Future research could replicate this study, using both a broader approach to recruitment and continuing recruitment efforts until a sample size and composition is reached that allows for a larger sample and potentially valuable subgroup analyses. We support and encourage people to adapt our methods and survey instrument to achieve this. This study developed a number of new measures for assessing the participants’ impact level of physical activity, as well as their healthcare preferences for information support and decision making. These items were carefully constructed, and the survey was informally pilot tested within a similar demographic prior to use; however, these items could be validated in future research. These limitations notwithstanding, and given the absence of other information, the results from this study can be used to guide the delivery of healthcare services and improve patient experiences.

## Conclusions

This research has important implications for the provision of healthcare for active young women with urinary incontinence. The results highlight the need for shared decision making between clinicians and patients, focusing on information such as cost, changes to daily habits, efficacy and discretion. These results could provide the basis for information fact sheets as well as decision-making tools such as patient decision aids, which should be available anonymously online. The results also highlight the need for easily accessible information and support, and it is suggested that general practitioners, physiotherapists or medical specialists should provide this information and support. Similar research with larger sample sizes is recommended, as this would enable comparisons in the healthcare preferences, priorities and experiences of different subgroups of young people with UI.

### Supplementary Information

Below is the link to the electronic supplementary material.

Supplementary file1 (PDF 378 KB)

Supplementary file2 (PDF 331 KB)

## Data Availability

The participants of this study did not consent to their data being shared publicly so research data are not available.
