# Supplementary material for: Urinary Incontinence in Active Female Young Adults: Healthcare Preferences, Priorities and Experiences
Source: Int Urogynecol J. 2024 May 7;35(6):1191–200. doi: 10.1007/s00192-024-05786-4 (PMC11245447; doi:10.1007/s00192-024-05786-4)
Supplement: Supplementary file 1 — Supplementary file1 (PDF 378 KB) [file 192_2024_5786_MOESM1_ESM.pdf]

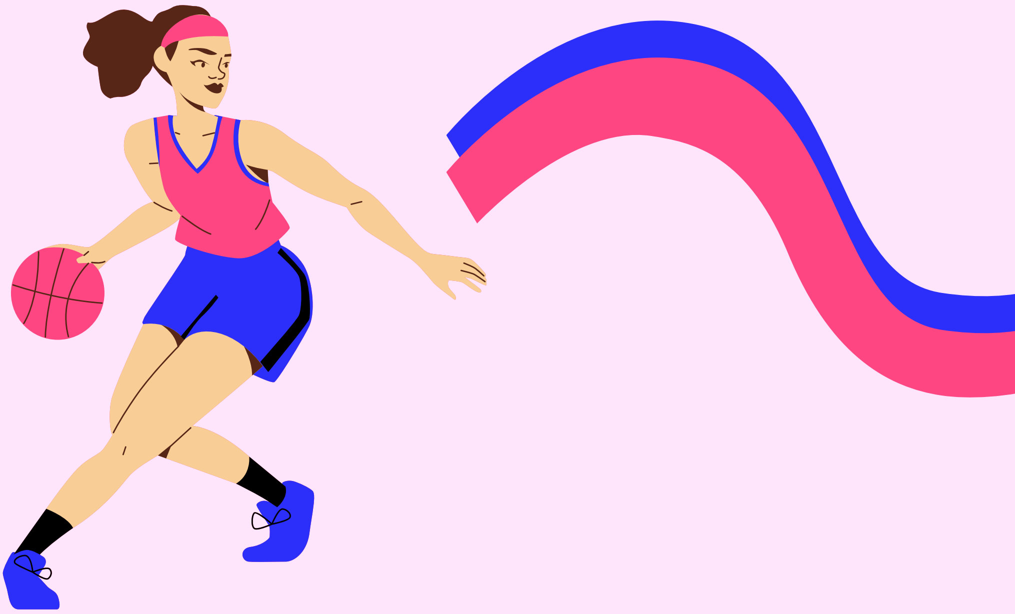

Do you sometimes leak urine when you exercise?

You may be eligible for our survey study to help people with urinary incontinence.

For more information visit:

www.theperiscopelab.org/youngpeople

THE UNIVERSITY OF SYDNEY

www.theperiscopelab .org/youngpeople

www.theperiscopelab .org/youngpeople
www.theperiscopelab .org/youngpeople
www.theperiscopelab .org/youngpeople
www.theperiscopelab .org/youngpeople
www.theperiscopelab .org/youngpeople
www.theperiscopelab .org/youngpeople
www.theperiscopelab .org/youngpeople
www.theperiscopelab .org/youngpeople

As many as 75% of female volleyball players sometimes leak urine.

If you are one of them you may be eligible to complete our survey.
www.theperiscopelab.org/youngpeople

THE UNIVERSITY OF SYDNEY

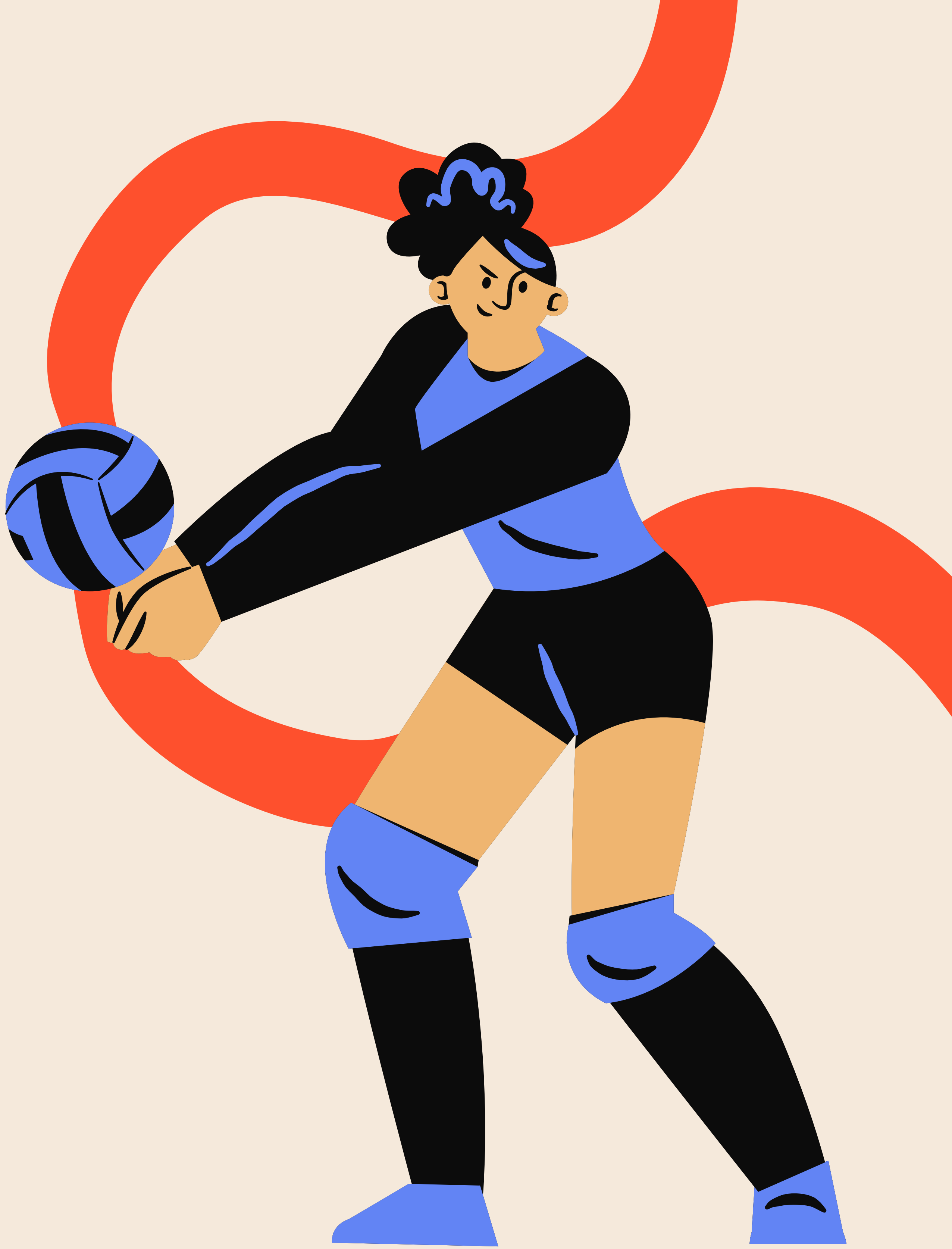

Do you sometimes leak urine when you exercise?

You may be eligible for our survey study to help people with urinary incontinence.

For more information visit:
www.theperiscopelab.org/athletes

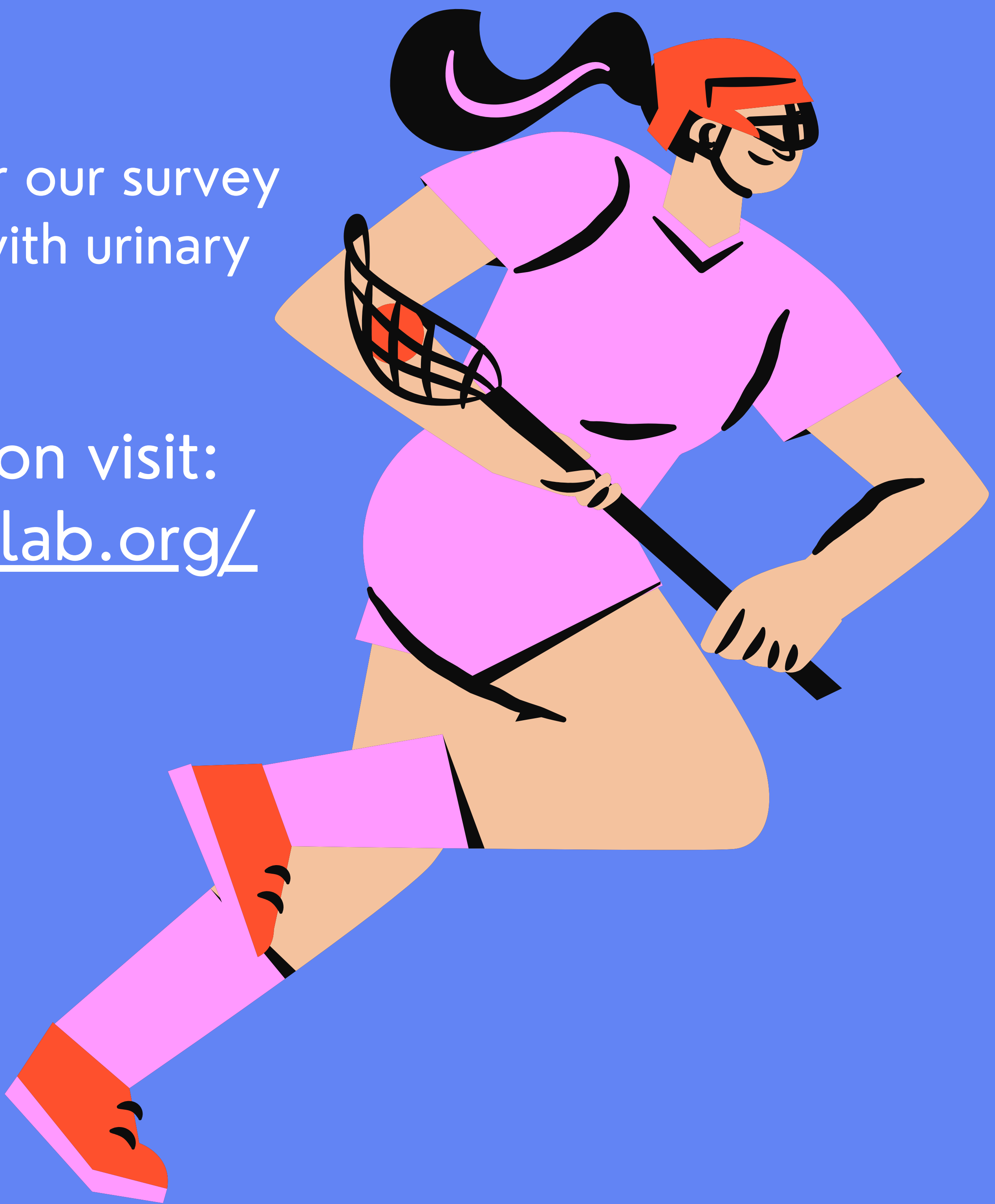

THE UNIVERSITY OF SYDNEY
