# Supplementary material for: Urinary Incontinence in Active Female Young Adults: Healthcare Preferences, Priorities and Experiences
Source: Int Urogynecol J. 2024 May 7;35(6):1191–200. doi: 10.1007/s00192-024-05786-4 (PMC11245447; doi:10.1007/s00192-024-05786-4)
Supplement: Supplementary file 2 — Supplementary file2 (PDF 331 KB) [file 192_2024_5786_MOESM2_ESM.pdf]

## Default Question Block

**Experiences and opinions of young female athletes with urinary incontinence**
Study Information Page

Dr Rachel Thompson (Head Researcher)
Email: rachel.thompson@sydney.edu.au | Phone: +61 2 8627 8073

Ms Rebecca Newark (Research Student)
Email: rnew4582@uni.sydney.edu.au

**1. What is this about?**
We are doing a study about the experiences and opinions of people who leak urine (wee). This study is being done as part of the Honours degree of research student, Rebecca Newark and will form part of the thesis submitted for this degree.

You do not have to take part in this study. This page will tell you more about the study so that you can make up your mind.

**2. Can I take part in this study?**
You can take part in this study if you:

1. were assigned female sex at birth,
2. are 18 to 30 years old,
3. live in Australia,

4. do regular physical activity, exercise, or sport,
5. have leaked urine at least once in the past four weeks, and
6. feel confident reading and writing in English.

**3. What will happen if I say yes?**
If you say yes, you will be asked to do an online survey. The survey is anonymous, which means we will not ask for your name. The survey includes questions about you, your experiences, and your preferences and opinions. For some questions you click an answer and for other questions you type an answer. We expect the survey to take 15-20 minutes.

If you have any questions about the study, you can contact us using the information at the top of the page. If you say yes and start the survey, we will take this to mean that you have decided to be in the study.

**4. What are the bad things about the study?**
Because some survey questions are personal in nature, it is possible that you may feel uncomfortable when doing the survey. If doing the survey raises any issues for you and you need information or support, you can contact:

- Lifeline Australia (13 11 14 or www.lifeline.org.au)
- Your General Practitioner (GP) or another health professional
- Health Direct (www.healthdirect.gov.au or 1800 022 222)

**5. What are the good things about the study?**
By being in this study you will be helping us to better understand people's experiences and find out what information and support they want. Once we have finished the study in early 2024, we will tell you what we found at this website.

**6. What are my rights?**
It is OK to skip questions if you are not comfortable answering them. It is also OK to discontinue the survey at any time by closing your browser. In this

situation, we will still use the information you have already provided.

What you tell us in the study is private. The only people that will see what you tell us are researchers involved in this study and people who work in information security at the University of Sydney. We will share what we found in this study in scientific articles, presentations, and materials for the public, but we won't include any information that could tell other people who you are.

**7. What if I am not happy about the study?**
If you are not happy with how we are doing the study and want to contact someone else, you can:

- **Call** the University of Sydney on +61 2 8627 8176
- **Email** the manager at human.ethics@sydney.edu.au

You can download a copy of this information to keep by clicking [here].

**Do you want to be in this study?**

| No | Yes |
|---|---|
| ◯ | ◯ |

**Eligibility 1**

**Do you feel confident reading and writing in English?**

| Yes | No |
|---|---|
| ◯ | ◯ |

**Eligibility 2**

## How old are you?

## Eligibility 3

## What sex were you assigned at birth on your original birth certificate?

- Female
- Male
- Other

## Eligibility 4

## What is your postcode?

- My postcode is:
- I do not live in Australia

## Eligibility 5

## How often do you do physical activity, exercise, or sport?

- Daily
- A few times a week
- A few times a month
- Less than a few times a month
- Never

## Eligibility 6

Many people leak urine some of the time. We are trying to find out how many people leak urine, and how much this bothers them.

We would be grateful if you could answer the following questions, thinking about how you have been, on average, over the PAST FOUR WEEKS.

**How often do you leak urine?**

- ◯ Never
- ◯ About once a week or less often
- ◯ Two or three times a week
- ◯ About once a day
- ◯ Several times a day
- ◯ All the time

**UI**

**We would like to know how much urine you think leaks.**
**How much urine do you usually leak (whether you wear protection or not)?**

- ◯ None
- ◯ A small amount
- ◯ A moderate amount
- ◯ A large amount

**Overall, how much does leaking urine interfere with your everyday life?**

Please select a number between 0 (not at all) and 10 (a great deal)

| 0 Not at all | 1 | 2 | 3 | 4 | 5 | 6 | 7 | 8 | 9 | 10 A great deal |
|---|---|---|---|---|---|---|---|---|---|---|
| ◯ | ◯ | ◯ | ◯ | ◯ | ◯ | ◯ | ◯ | ◯ | ◯ | ◯ |

**When does urine leak?**

Please select all that apply to you

- ☐ Never – urine does not leak
- ☐ Leaks before you can get to the toilet
- ☐ Leaks when you cough or sneeze
- ☐ Leaks when you are asleep
- ☐ Leaks when you are physically active/exercising
- ☐ Leaks when you have finished urinating and are dressed
- ☐ Leaks for no obvious reason
- ☐ Leaks all the time

**Have you ever talked to a health professional about your leaking urine?**

| Yes | No |
|---|---|
| ○ | ○ |

**What, if anything, have you tried or used to manage your leaking urine?**

**Which people or places would you like to receive information and support about leaking urine from?**

Please select all that apply

- ☐ General practitioner (GP)

- [ ] Physiotherapist
- [ ] Medical specialist (e.g., urogynecologist)
- [ ] Sports coach
- [ ] Family or friend
- [ ] A website
- [ ] Social media
- [ ] ______ Another health professional. Please describe:
- [ ] ______ Another person. Please describe:
- [ ] ______ Other. Please describe:
- [ ] None of these

**These are the people or places you said you would like to receive information and support from about leaking urine.**

**Which three people or places would you most like to receive information and support from?**

Please drag your them into the box on the right.

| Items | My Top 3 |
|---|---|
| » General practitioner (GP) | |
| » Physiotherapist | |
| » Medical specialist (e.g., urogynecologist) | |
| » Sports coach | |
| » Family or friend | |
| » A website | |
| » Social media | |
| » Another health professional. Please describe: ______ | |

» Another person. Please describe:

» Other. Please describe:

» None of these

**How would you like to receive information and support about leaking urine?**

Please select all that apply

- ☐ In person, one-on-one
- ☐ In person, in a group
- ☐ Telephone call
- ☐ Video call
- ☐ Text message
- ☐ Online, without giving my name
- ☐ Online, giving my name
- ☐ Other. Please describe:

**There are lots of options for managing leaking urine.**

**If you were deciding how to manage your leaking urine, what would you want to know about each option?**

Please select all that apply

- ☐ Does it involve changing my habits or activities (e.g., how much coffee I drink)?
- ☐ Will it stop me leaking urine completely?
- ☐ How much does it cost?
- ☐ Will it reduce how often or how much I leak urine?
- ☐ What are the risks or side effects?

- [ ] Could it make my leaking urine worse?
- [ ] Does it involve seeing a health professional face-to-face?
- [ ] How does it work?
- [ ] Does it involve a health professional touching inside or near my vagina?
- [ ] Does it involve me putting something into my vagina?
- [ ] Does it involve having surgery (an operation)?
- [ ] Does it involve staying in a hospital?
- [ ] Can I stop using it if I change my mind?
- [ ] Does it involve having a needle?
- [ ] Does it involve a device being put in my body?
- [ ] How long does it take to start working?
- [ ] Will other people know that I am using it?
- [ ] Has it been properly studied?
- [ ] Can I access it online?
- [ ] Does it require regular visits to a health professional?
- [ ] ________ Other:
- [ ] ________ Other:
- [ ] ________ Other:

**These are the things you said you would want to know about options for managing leaking urine.**

**Which three things are most important to you?**

Please drag your them into the box on the right.

| Items | My Top 3 |
| --- | --- |
| » Does it involve changing my habits or activities (e.g., how much coffee I drink)? | |
| » Will it stop me leaking urine completely? | |

» How much does it cost?

» Will it reduce how often or how much I leak urine?

» What are the risks or side effects?

» Could it make my leaking urine worse?

» Does it involve seeing a health professional face-to-face?

» How does it work?

» Does it involve a health professional touching inside or near my vagina?

» Does it involve me putting something into my vagina?

» Does it involve having surgery (an operation)?

» Does it involve staying in a hospital?

» Can I stop using it if I change my mind?

» Does it involve having a needle?

» Does it involve a device being put in my body?

» How long does it take to start working?

» Will other people know that I am using it?

» Has it been properly studied?

» Can I access it online?

» Does it require regular visits to a health professional?

» Other:

» Other:

» Other:

## If you were deciding how to manage your leaking urine, how would you want the final decision to be made?

- ◯ I would want to make the final decision
- ◯ I would want to make the final decision after seriously considering my health professional's opinion
- ◯ I would want to share responsibility for the final decision with my health professional
- ◯ I would want my health professional to make the final decision after seriously considering my opinion
- ◯ I would want my health professional to make the final decision

## Characteristics

## What type of physical activity, exercise, or sport do you do most often?

**What is the impact level of the physical activity, exercise, or sport you do most often?**

Low impact activities are things like walking or swimming.
High impact activities are things like volleyball or gymnastics

| 0 Low impact | 1 | 2 | 3 | 4 | 5 | 6 | 7 | 8 | 9 | 10 High impact |
|---|---|---|---|---|---|---|---|---|---|---|
| ○ | ○ | ○ | ○ | ○ | ○ | ○ | ○ | ○ | ○ | ○ |

**Which of these best describes the physical activity, exercise, or sport you do most often?**

- ○ Recreational
- ○ Regional level
- ○ State level
- ○ National level
- ○ Elite (paid)

**Socio-demographics**

**Which of the following describe you?**

Please select all that apply

- ☐ Aboriginal
- ☐ Torres Strait Islander
- ☐ None of the above

**What language(s) do you speak at home?**

Please select all that apply

- ◯ English
- ◯ [ ] Other:

**Which of these apply to you?**

Please select all that apply

- ☐ I have been pregnant
- ☐ I have had one baby
- ☐ I have had more than one baby
- ☐ None of the above

**How confident are you filling out medical forms by yourself?**

| Extremely | Quite a bit | Somewhat | A little bit | Not at all |
|---|---|---|---|---|
| ◯ | ◯ | ◯ | ◯ | ◯ |

**Please use this space if there is anything else you would like to tell us about your experiences or opinions.**

Powered by Qualtrics
